# Human papillomavirus types in non-cervical high-grade intraepithelial neoplasias and invasive carcinomas from San Luis Potosí, Mexico: a retrospective cross-sectional study

**DOI:** 10.1186/s13027-015-0027-8

**Published:** 2015-09-28

**Authors:** Claudia Magaña-León, Cuauhtémoc Oros, Rubén López-Revilla

**Affiliations:** División de Biología Molecular, Instituto Potosino de Investigación Científica y Tecnológica, Camino a la Presa San José 2055, 78216 San Luis Potosí, S.L.P. Mexico; Departamento de Patología, Hospital Central Ignacio Morones Prieto, Av. Venustiano Carranza 2395, 78240 San Luis Potosí, S.L.P. Mexico

**Keywords:** HPV, Non-cervical carcinomas, SPF10, INNO-LiPA

## Abstract

**Background:**

Viral infections and the burden of high-grade intraepithelial neoplasias (HIN) and invasive carcinomas (IC) associated to infections by human papillomavirus (HPV) types may be prevented by type-specific anti-HPV vaccines. This study determined the prevalence of HPV types in non-cervical HIN and IC diagnosed from 1999 to 2011 at a general hospital in San Luis Potosí, Mexico.

**Methods:**

Review of the 67 formaldehyde-fixed paraffin-embedded non-cervical specimens initially diagnosed as HIN (*n* = 28) or IC (*n* = 39) confirmed the presence of tumor tissue in 63 of them and changed the diagnosis of 24 from HIN to low-grade intraepithelial neoplasias, that were excluded from the study. HPV DNA was detected with the SPF10-DNA enzyme immunoassay in the 39 cases included, and viral types in the HPV-positive tumors were identified with the INNO-LiPA linear probe array.

**Results:**

Among the cases included, four HIN were located in the vagina (*n* = 3) and vulva (*n* = 1), and 35 IC in the oral cavity (*n* = 19), penis (*n* = 8), vagina (*n* = 7) and vulva (*n* = 1). There were 13 HPV-positive cases from the vagina (*n* = 7), vulva (*n* = 1), penis (*n* = 1) and oral cavity (*n* = 1). The viral types identified were the high-risk types HPV16 in the vagina (*n* = 3) and vulva (*n* = 3), HPV45 in the vagina (*n* = 2), HPV59 in the vagina (*n* = 1) and penis (*n* = 1), HPV33 in the vagina (*n* = 1),and HPV35 in the tongue(*n* = 1); and the low-risk types HPV54 in the vagina (*n* = 1), and HPV11 in the vulva (*n* = 1).

**Conclusions:**

Five high-risk viral types (HPV16, 45, 59, 33 and 35) and two low-risk types (HPV11 and 54) infect one third of the non-cervical HIN and IC included. Most infections are by a single HPV high-risk type, the most prevalent one being HPV16. Vagina is the most frequent location of the HPV-positive tumors. Vaccination against HPV16 and HPV18 could have prevented around half of the HPV-positive tumors.

## Background

The order of prevalence of high-risk HPV types associated to cervical carcinomas around the world is HPV16, 18, 31, 33, 35, 45, 52 and 58 [1]. It is similar for cervical carcinomas in Mexico [2–4] and abnormal cervical scrapes in the Mexican states of San Luis Potosí and Guanajuato [5].

Persistent infection with high-risk HPV types is necessary for neoplastic transformation of the normal cervical epithelium to high-grade intraepithelial neoplasia (HIN) and its progression to invasive carcinoma (IC) [6–8]. Viral infection and the burden of neoplastic lesions may be reduced through prophylactic HPV vaccination [9], but HPV type distribution has to be determined to establish health care policies and vaccination programs in each area [1, 10].

Association of HPV types to non-cervical carcinomas has been demonstrated in recent years. HPV16, 33 and 18 are the most prevalent types in vulvar carcinomas [11, 12], HPV16, 18, 31 and 33 in vaginal and anal carcinomas [13], and HPV16 in oral carcinomas [14]. In Mexico, Flores de la Torre *et al.* [15] found HPV DNA in nearly half of 117 head and neck carcinomas with HPV16 as the major viral type.

In this study we identified the HPV types present in HIN and IC from the oral cavity, penis, vagina and vulva diagnosed in a 12-year period at the largest general hospital of San Luis Potosí, to estimate the effect that anti-HPV vaccination could have had to prevent them.

## Materials and methods

### Study design and population

The protocol of this work was based on that of a multicentric study designed by the ICO and approved by the Delft Diagnostic Laboratory (DDL) [1]. The study protocol and consent procedure were approved by the Research and Ethics Committees of the Hospital Central Ignacio Morones Prieto and the Institut Català d’Oncologia (ICO). Like similar studies, its implementation did not require informed consent from the patients, who are not identified by name in accordance with the Mexican General Health Law.

The study considered the 67 non-cervical tumors whose formalin-fixed paraffin-embedded (FFPE) blocks were recovered. The initial IC and HIN diagnoses were performed between 1999 and 2011 at the Pathology Department of Hospital Central Ignacio Morones Prieto.

### Selection and analysis of the cases included

The cases included were those in which the ICO Pathology Reference Laboratory confirmed the presence of tumor tissue and made the final (IC or HIN) histopathological diagnoses. FFPE blocks from the 67 cases were analyzed by the sandwich method based on five sections [1]. Section 1 (negative control), 5 μm thick, was obtained from a virgin paraffin block and placed in a tube. The following four serial sections were from the paraffin block of an acceptable case (*i.e.*, containing tumor tissue); sections 2 and 5, 3 μm thick, were placed on individual microscope slides; sections 3 and 4, 5 μm thick, were placed in each of two tubes. In order to avoid cross contamination the microtome blade was replaced after obtaining the sections from each tumor block. Sections on the slides were stained with hematoxylin-eosin and subjected to histopathological review; DNA was extracted from sections 1 and 3 or 5 for HPV detection and typing.

The cases were excluded if slides 2 and 4 did not contain tumor tissue. When only the first slide contained tumor tissue, DNA extracted from tube 1 was amplified using the SPF10 (short PCR fragment generated with 10 oligonucleotides) method followed by DNA enzyme immunoassay (DEIA) [12] to detect HPV sequences. DNA extracted from tube 2 was amplified when tumor tissue was found in both slides or only in the second one [1].

### DNA extraction and HPV detection and typing

Each 5 μm section from a paraffin block with tumor tissue was mixed with 250 μL of proteinase K (1 mg/mL) dissolved in 10 mM Tris–HCl, 1 mM EDTA (pH 8.0) and 0.5 % Tween-20, and incubated overnight at 60 °C. Proteinase K was then inactivated by heating at 95 °C for 10 min. To determine the presence of HPV DNA, 1 μL of the supernatant from each section treated with proteinase K was added to the SPF10-DEIA PCR mix under the conditions described by Kleter *et al.* [17].

Samples of the HPV-positive DNA extracts were analyzed with the INNO-LiPA (linear probe array) on-strip reverse hybridization method (Innogenetics, DDL) which identifies 25 of the 40 anogenital HPV types [18].

## Results

### Stages of the study

**Fig. 1:**
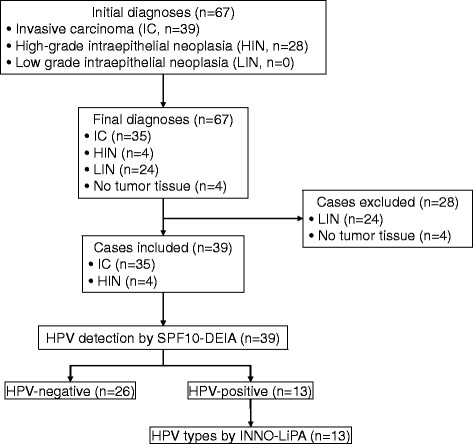
Stages of the study. Histopathological review showed that four of the 67 initial paraffin blocks lacked tumor tissue and were discarded. Of the 63 acceptable paraffin blocks, 28 were excluded because their initial diagnoses changed from HIN to LIN. DNA extracted from the paraffin blocks of the 39 cases included (35 IC and four HIN) was subjected to the SPF10-DEIA assay. The 13 HPV-positive cases were further analyzed with the INNO-LiPA assay to identify the viral types

The study was carried out in five steps (Fig. 1): 1) 67 formalin-fixed paraffin-embedded (FFPE) blocks from non-cervical tumors with initial HIN or IC diagnoses were retrieved from the Pathology Department of Hospital Central Ignacio Morones Prieto; 2) sections were obtained by the sandwich method and final diagnoses were performed at the ICO Pathology Reference Laboratory; 3) cases with final IC or HIN histopathological diagnoses were included, and those lacking tumor tissue or with final diagnoses of low-grade intraepithelial neoplasia (LIN) were excluded; 4) SPF10-DEIA was performed to identify HPV-positive and -negative tumors in the cases included; 5) viral types in the HPV-positive cases were identified with the INNO-LiPA assay.

### Histopathological diagnosis and case selection

Initial diagnoses of the 63 acceptable cases were 28 HIN and 35 IC (Table 1). There were 27 HIN located in female genitalia (25 in the vagina, two in the vulva) and one in the anus. Nineteen IC were located in the oral cavity (nine in the larynx, six in the tongue, two in the palate, one in the jaw, one in the pharynx), eight in the penis, eight in female genitalia (seven in the vagina, one in the vulva).

**Table 1:** Anatomic location and histopathological diagnosis of the tumors

Location		Diagnosis^a^				
		Initial		Final^b^		
		HIN	IC	LIN	HIN	IC
Anogenital	Vulva	2	1	1	1	1
	Vagina	25	7	22	3	7
	Anus	1	0	1	0	0
	Penis	0	8	0	0	8
	Subtotal	28	16	24	4	16
Oral	Larynx	0	9	0	0	9
	Tongue	0	6	0	0	6
	Palate	0	2	0	0	2
	Jaw	0	1	0	0	1
	Pharynx	0	1	0	0	1
	Subtotal	0	19	0	0	19
	Total	28	35	24	4	35

^a^ *IC* invasive carcinoma, *HIN* high-grade intraepithelial neoplasia, *LIN* low-grade intraepithelial neoplasia

^b^The study excluded the 24 cases with final LIN diagnoses and included the 39 cases with final HIN and IC diagnoses

Review of the slides showed that four FFPE blocks lacked tumor tissue and led to the final diagnoses which determined the inclusion or exclusion of the tumor-containing specimens. Twenty-four cases with initial HIN diagnoses changed to low-grade intraepithelial neoplasia (LIN) and therefore excluded (22 from the vagina, one from the vulva and one from the anus) (Table 1).

Agreement between the initial and final diagnoses for IC was 100 % (35/35), but only 14.3 % (4/28) for HIN (Table 1). The 39 cases included were four HIN (three from the vagina, one from the vulva) and all 35 IC from the already mentioned locations.

### HPV detection and typing

Fourteen of the cases included were HPV-positive (35.9 %), comprising 10 IC (six from the vagina, one from the vulva, one from the penis and one from the tongue), and four HIN (three from the vagina and one from the vulva).

Seven viral types were identified with the INNO-LiPA assay applied to the HPV-positive cases (Table 2): five of high-risk (HPV16, 45, 59, 33 and 35) and two of low-risk (HPV11 and 54). The most prevalent type was HPV16, present in six cases (two vaginal and one vulvar IC, and three vaginal HIN), followed by HPV45 in two vaginal IC, and HPV59 in two IC (one of the vagina and one of the penis). There were five cases with each of the following types: HPV33 (vaginal IC), HPV35 (lingual IC), HPV54 (vaginal IC) and HPV11 (vulvar HIN) (Table 2).

**Table 2:** Viral types identified in the HIN and IC cases analyzed for HPV

Cases	HIN		IC								Total
	Vagina	Vulva	Larynx	Tongue	Palate	Jaw	Pharynx	Penis	Vagina^a^	Vulva	
Analyzed for HPV, n (%)	3 (7.7)	1 (2.6)	9 (23.1)	6 (15.4)	2 (5.1)	1 (2.6)	1 (2.6)	8 (20.5)	7 (17.9)	1 (2.6)	39 (100.0)
HPV type detected, n (%)											
16	3	0	0	0	0	0	0	0	2	1	6 (15.4)
59	0	0	0	0	0	0	0	1	1	0	2 (5.1)
45	0	0	0	0	0	0	0	0	2	0	2 (5.1)
54	0	0	0	0	0	0	0	0	1	0	1 (2.6)
35	0	0	0	1	0	0	0	0	0	0	1 (2.6)
33	0	0	0	0	0	0	0	0	1	0	1 (2.6)
11	0	1	0	0	0	0	0	0	0	0	1 (2.6)
HPV positive cases, n (%)	3 (7.7)	1 (2.6)	0 (0.0)	1 (2.6)	0 (0.0)	0 (0.0)	0 (0.0)	1 (2.6)	7 (17.9)	1 (2.6)	14 (35.9)

^a^A vaginal IC was the only case with a double infection, by HPV16 and HPV59

Twelve of the 13 HPV-positive cases (92.3 %) had DNA from a single high-risk type. The only vaginal IC had a double infection by a high-risk and a low-risk type (HPV16 and HPV59). The only vulvar HIN had the low-risk HPV11 type.

## Discussion

The SPF10-DEIA-INNOLiPA assay used in this work is highly sensitive and specific for HPV DNA detection and typing. It has been used in several multicenter studies [1, 12] because it generates 65-base-pair-long viral amplicons from the DNA extracted from FFPE sections, whose fixation and manipulation damage DNA and prevent the generation of longer amplicons [17, 19].

The presence of tumor tissue was confirmed in 63 out of the 67 original paraffin blocks and the study included only 39 cases with final HIN or IC diagnoses because the initial diagnoses changed from HIN to LIN in 24 cases. The initial and final diagnoses coincided in all the 35 IC cases included, whereas 24 of the 28 initial HIN cases changed to LIN and were excluded. Since premalignant vaginal squamous epithelial lesions are classified as grade 1 (LIN), grade 2 and grade 3 (the last two grades synonymous with HIN) [20], the diagnostic discordance appears to result from having included grade 2 and 3 lesions in the group of initial diagnoses and grade 3 lesions in the group of final diagnoses. On the other hand, the exclusion of 24 initial HIN cases —22 from the vagina— improved the quality of the study.

The more stringent diagnostic criteria of the ICO Pathology Reference Laboratory for preinvasive neoplastic lesions resulted in the inclusion of all the initially diagnosed IC but only 14.3 % of the initially diagnosed HIN.

LIN samples were excluded from the study because HPV infections are spontaneously cleared in most of them, while they persist in HIN and IC [21]. Most of the cases included were IC (89.7 %) with the following anatomic locations: 48.7 % in the oral cavity (larynx 23.1 %, tongue 15.4 %, palate 5.1 %, jaw 2.6 %, pharynx 2.6 %); 41.0 % in male and female genitalia (penis 20.5 %, 17.9 % vagina, vulva 2.6 %). The HIN cases included were located in female genitalia (10.3 %): vagina (7.7 %) and vulva (2.6 %).

Only one third of the cases included were HPV-positive. Nine were IC: six from the vagina, one from the vulva, one from the penis and one from the oral cavity; four were HIN: three from the vagina and one from the vulva. These results are consistent with those of several multicenter studies; Kreimer *et al.* [22] showed an HPV prevalence around 25 % in head and neck carcinomas, De Vuyst *et al.* [13] found prevalences of 85.3 % for IC and 40.4 % for HIN in the vulva and of 90.1 % for IC and 69.9 % for HIN in the vagina, and Van Aar *et al.* [23] of 16 to 32 % in penile carcinomas.

Seven viral types were identified in the HPV-positive cases: five of high risk (HPV16, 45, 59, 33, and 35), and two of low-risk (HPV11 in a vulvar HIN and HPV54 in a vaginal IC). Infections with only one low-risk HPV type have been shown in vulvar IC (HPV6, 26 or 61) by Sutton *et al.* [11], and in anal and perineal HIN and IC (HPV6 and 11) by Cornall *et al.* [24].

The most prevalent viral type is HPV16 (46.1 %), followed by HPV45 and 59 (15.4 % each). These data are consistent with HPV16 being the most prevalent type in vulvar, vaginal, anal and oral carcinomas [13, 22].

HPV35 was identified in the only oral IC despite the fact that HPV18 is often associated with non-cervical carcinomas [25] and HPV16 is the most prevalent in oral carcinomas [14].

A single viral type was detected in 12 of the 13 HPV-positive tumors (92.3 %), and two types in the remaining one. This finding suggest that most infections by a single HPV type are linked to tumor development and agree with a multicenter study which demonstrated single infections in 93 % in cervical and non-cervical HPV-positive carcinomas [1].

The small sample size resulting from the low prevalence of the non cervical carcinomas is an obvious limitation of the study, which includes the corresponding tumors diagnosed for over a decade at a large Mexican hospital, identifies the HPV-positive tumors and the viral types to which they can be attributed, and calculates the ones could that have been prevented by anti-HPV vaccination.

The 13 HPV-positive tumors were, in descending order of frequency, nine vaginal, two vulvar, one penile, and one lingual. Assuming that the divalent, quadrivalent and nonavalent HPV vaccines induce complete protection but only against the viral types to which they are directed, the divalent and quadrivalent vaccines could have prevented seven tumors (~54 %) and the nonavalent vaccine 11 tumors (~85 %), mostly vaginal and vulvar.

## Conclusions

Final histopathological diagnoses leading to the inclusion or exclusion of non-cervical tumors agreed with all the initial IC diagnoses but only one-seventh of the initial HIN diagnoses. Five high-risk viral types (HPV16, 45, 59, 33 and 35) and two low-risk types (HPV11 and 54) infect one third of the tumors included. Twelve of the 13 HPV-positive tumors (92.3 %) are infected by a single viral type. The most prevalent type is HPV16 (46.1 %), followed by HPV45 and 59 (15.4 % each). Vagina is the predominant location of the HPV-positive tumors. Vaccination with the divalent, quadrivalent or nonavalent HPV vaccines in this region could have prevented from half to over two thirds of the HPV-positive non-cervical tumors, mostly vaginal.

## Abbreviations

CONACYT: National Council for Science and Technology (Mexico)
DDL: Delft Diagnostic Laboratory (Voorburg, Netherlands)
DEIA: DNA enzyme immunoassay
FFPE: Formalin-fixed paraffin-embedded
HIN: High-grade intraepithelial neoplasia
HPV: Human papillomavirus
IC: Invasive carcinoma
ICO: Institut Català d’Oncologia (Barcelona, Spain)
LIN: Low-grade intraepithelial neoplasia
LiPA: Linear probe array
SPF10: Short PCR fragment generated with 10 oligonucleotides
