# Erratum to: Human papillomavirus types in non-cervical high-grade intraepithelial neoplasias and invasive carcinomas from San Luis Potosí, Mexico: a retrospective cross-sectional study

**DOI:** 10.1186/s13027-016-0048-y

**Published:** 2016-02-25

**Authors:** Claudia Magaña-León, Cuauhtémoc Oros, Rubén López-Revilla

**Affiliations:** División de Biología Molecular, Instituto Potosino de Investigación Científica y Tecnológica, Camino a la Presa San José 2055, San Luis Potosí, 78216 S.L.P. Mexico; Departamento de Patología, Hospital Central Ignacio Morones Prieto, Av. Venustiano Carranza 2395, San Luis Potosí, 78240 S.L.P. Mexico

## Erratum

After publication of this article [1], the authors noticed that the references listed had not been published according to the style required for *Infectious Agents and Cancer*.

The reference list which was published in the original article [1] is listed below:

1. Bosch FX, Lorincz A, Munoz N, Meijer CJLM, Shah KV. The causal relation between human papillomavirus and cervical cancer. J Clin Pathol. 2002;55:244–65.
2. Bruni L, Diaz M, Castellsague X, Ferrer E, Bosch FX, de Sanjose S. Cervical human papillomavirus prevalence in 5 continents: meta-analysis of 1 million women with normal cytological findings. J Infect Dis. 2010;202:1789–99.
3. Cornall AM, Roberts JM, Garland SM, Hillman RJ, Grulich AE, Tabrizi SN. Anal and perianal squamous carcinomas and high-grade intraepithelial lesions exclusively associated with “low-risk” HPV genotypes 6 and 11. Int J Cancer. 2013;133:2253–8.
4. D'Souza G, Kreimer AR, Viscidi R, Pawlita M, Fakhry C, Koch WM, et al. Case–control study of human papillomavirus and oropharyngeal cancer. N Engl J Med. 2007;356:1944–56.
5. de Sanjose S, Alemany L, Ordi J, Tous S, Alejo M, Bigby SM, et al. Worldwide human papillomavirus genotype attribution in over 2000 cases of intraepithelial and invasive lesions of the vulva. Eur J Cancer. 2013;49:3450–61.
6. de Sanjose S, Quint WG, Alemany L, Geraets DT, Klaustermeier JE, Lloveras B, et al. Human papillomavirus genotype attribution in invasive cervical cancer: a retrospective cross-sectional worldwide study. Lancet Oncol. 2010;11:1048–56.
7. De Vuyst H, Clifford GM, Nascimento MC, Madeleine MM, Franceschi S. Prevalence and type distribution of human papillomavirus in carcinoma and intraepithelial neoplasia of the vulva, vagina and anus: a meta-analysis. Int J Cancer. 2009;124:1626–36.
8. Flores-De La Torre C, Hernandez-Hernandez DM, Gallegos-Hernandez JF. Human papilloma virus in patients with epidermoid head and neck carcinoma: a prognostic factor? Cirugia y Cirujanos. 2010;78:221–8.
9. Garland SM, Smith JS. Human papillomavirus vaccines: current status and future prospects. Drugs. 2010;70:1079–98.
10. Iarc. Human Papillomaviruses. IARC monographs on the evaluation of carcinogenic risks to humans. 2005. p. 90.
11. Illades-Aguiar B, Alarcon-Romero Ldel C, Antonio-Vejar V, Zamudio-Lopez N, Sales-Linares N, Flores-Alfaro E, et al. Prevalence and distribution of human papillomavirus types in cervical cancer, squamous intraepithelial lesions, and with no intraepithelial lesions in women from Southern Mexico. Gynecol Oncol. 2010;117:291–6.
12. Kleter B, van Doorn LJ, Schrauwen L, Molijn A, Sastrowijoto S, ter Schegget J, et al. Development and clinical evaluation of a highly sensitive PCRreverse hybridization line probe assay for detection and identification of anogenital human papillomavirus. J Clin Microbiol. 1999;37:2508–17.
13. Kleter B, van Doorn LJ, Schrauwen L, Molijn A, Sastrowijoto S, ter Schegget J, et al. Development and clinical evaluation of a highly sensitive PCRreverse hybridization line probe assay for detection and identification of anogenital human papillomavirus. J Clin Microbiol. 1999;37(8):2508-17. Magaña-León et al. Infectious Agents and Cancer (2015) 10:33 Page 5 of 6
14. Kleter B, van Doorn LJ, ter Schegget J, Schrauwen L, van Krimpen K, Burger M, et al. Novel short-fragment PCR assay for highly sensitive broad-spectrum detection of anogenital human papillomaviruses. The American journal of pathology. 1998;153(6):1731-9. doi:10.1016/S0002-9440(10)65688-X.
15. Kleter B, van Doorn LJ, Schrauwen L, Molijn A, Sastrowijoto S, ter Schegget J, et al. (1999) Development and clinical evaluation of a highly sensitive PCR-reverse hybridization line probe assay for detection and identification of anogenital human papillomavirus. Journal of clinical microbiology 37:2508-2517
16. Kleter B, van Doorn LJ, ter Schegget J, Schrauwen L, van Krimpen K, Burger M, et al. (1998) Novel short-fragment PCR assay for highly sensitive broadspectrum detection of anogenital human papillomaviruses. The American journal of pathology 153:1731-1739.
17. Lizano M, De la Cruz-Hernandez E, Carrillo-Garcia A, Garcia-Carranca A, Ponce de Leon-Rosales S, Duenas-Gonzalez A, et al. Distribution of HPV16 and 18 intratypic variants in normal cytology, intraepithelial lesions, and cervical cancer in a Mexican population. Gynecol Oncol. 2006;102:230–5.
18. Montoya-Fuentes H, Suarez Rincon AE, Ramirez-Munoz MP, ArevaloLagunas I, Moran Moguel MC, Gallegos Arreola MP, et al. The detection of human papillomavirus 16, 18, 35 and 58 in cervical-uterine cancer and advanced degree of squamous intraepithelial lesions in Western Mexico: clinical-molecular correlation. Ginecol Obstet Mex. 2001;69:137–42.
19. Schiffman M, Castle PE, Jeronimo J, Rodriguez AC, Wacholder S. Human papillomavirus and cervical cancer. Lancet. 2007;370:890–907.
20. Smith JS, Lindsay L, Hoots B, Keys J, Franceschi S, Winer R, et al. Human papillomavirus type distribution in invasive cervical cancer and high-grade cervical lesions: a meta-analysis update. Int J Cancer. 2007;121:621–32.
21. Sutton BC, Allen RA, Moore WE, Dunn ST. Distribution of human papillomavirus genotypes in invasive squamous carcinoma of the vulva. Mod Pathol. 2008;21:345–54.
22. Tavassoli FA, Devilee P. Tumours of the vagina. Epithelial tumours. In: Andersen ES, Paavonen J, Murnahan M, Östör AG, Hanselaar AG, Bergeron C, Dobbs SP, editors. Pathology and Genetics of Tumours of the Breast and Female Genital Organs. Lyon: IARC Press; 2003. p. 293–301.
23. Van Aar F, Mooij SH, Van Der Sande MA, Speksnijder AG, Stolte IG, Meijer CJ, et al. Anal and penile high-risk human papillomavirus prevalence in HIV-negative and HIV-infected MSM. Aids. 2013.
24. Walboomers JMM, Jacobs MV, Manos MM, Bosch FX, Kummer JA, Shah KV, et al. Human papillomavirus is a necessary cause of invasive cervical cancer worldwide. J Pathol. 1999;189:12–9.

The correct reference list is included below and the original article [1] has been updated to reflect the current reference list.

1. de Sanjose S, Quint WG, Alemany L, Geraets DT, Klaustermeier JE, Lloveras B et al. Human papillomavirus genotype attribution in invasive cervical cancer: a retrospective cross-sectional worldwide study. Lancet Oncol. 2010;11(11):1048-56. doi:10.1016/S1470-2045(10)70230-8 S1470-2045(10)70230-8 [pii].
2. Illades-Aguiar B, Alarcon-Romero Ldel C, Antonio-Vejar V, Zamudio-Lopez N, Sales-Linares N, Flores-Alfaro E et al. Prevalence and distribution of human papillomavirus types in cervical cancer, squamous intraepithelial lesions, and with no intraepithelial lesions in women from Southern Mexico. Gynecol Oncol. 2010;117(2):291-6. doi:10.1016/j.ygyno.2010.01.036.
3. Lizano M, De la Cruz-Hernandez E, Carrillo-Garcia A, Garcia-Carranca A, Ponce de Leon-Rosales S, Duenas-Gonzalez A et al. Distribution of HPV16 and 18 intratypic variants in normal cytology, intraepithelial lesions, and cervical cancer in a Mexican population. Gynecologic oncology. 2006;102(2):230-5. doi:10.1016/j.ygyno.2005.12.002.
4. Montoya-Fuentes H, Suarez Rincon AE, Ramirez-Munoz MP, Arevalo-Lagunas I, Moran Moguel MC, Gallegos Arreola MP et al. [The detection of human papillomavirus 16, 18, 35 and 58 in cervical-uterine cancer and advanced degree of squamous intraepithelial lesions in Western Mexico: clinical-molecular correlation]. Ginecol Obstet Mex. 2001;69:137-42.
5. Lopez-Revilla R, Martinez-Contreras LA, Sanchez-Garza M. Prevalence of high-risk human papillomavirus types in Mexican women with cervical intraepithelial neoplasia and invasive carcinoma. Infect Agent Cancer. 2008;3:3. doi:10.1186/1750-9378-3-3.
6. Bosch FX, Lorincz A, Munoz N, Meijer CJLM, Shah KV. The causal relation between human papillomavirus and cervical cancer. Journal of Clinical Pathology. 2002;55(4):244-65.
7. Schiffman M, Castle PE, Jeronimo J, Rodriguez AC, Wacholder S. Human papillomavirus and cervical cancer. Lancet. 2007;370(9590):890-907. doi:Doi 10.1016/S0140-6736(07)61416-0.
8. Walboomers JMM, Jacobs MV, Manos MM, Bosch FX, Kummer JA, Shah KV et al. Human papillomavirus is a necessary cause of invasive cervical cancer worldwide. Journal of Pathology. 1999;189(1):12-9. doi:Doi 10.1002/(Sici)1096-9896(199909)189:1%3C12::Aid-Path431%3E3.0.Co;2-F.
9. Garland SM, Smith JS. Human papillomavirus vaccines: current status and future prospects. Drugs. 2010;70(9):1079-98. doi:10.2165/10898580-000000000-000001 [pii].
10. Bruni L, Diaz M, Castellsague X, Ferrer E, Bosch FX, de Sanjose S. Cervical human papillomavirus prevalence in 5 continents: meta-analysis of 1 million women with normal cytological findings. J Infect Dis. 2010;202(12):1789-99. doi:10.1086/657321.
11. Sutton BC, Allen RA, Moore WE, Dunn ST. Distribution of human papillomavirus genotypes in invasive squamous carcinoma of the vulva. Modern pathology : an official journal of the United States and Canadian Academy of Pathology, Inc. 2008;21(3):345-54. doi:10.1038/modpathol.3801010.
12. de Sanjose S, Alemany L, Ordi J, Tous S, Alejo M, Bigby SM et al. Worldwide human papillomavirus genotype attribution in over 2000 cases of intraepithelial and invasive lesions of the vulva. Eur J Cancer. 2013;49(16):3450-61. doi:10.1016/j.ejca.2013.06.033.
13. De Vuyst H, Clifford GM, Nascimento MC, Madeleine MM, Franceschi S. Prevalence and type distribution of human papillomavirus in carcinoma and intraepithelial neoplasia of the vulva, vagina and anus: a meta-analysis. International journal of cancer Journal international du cancer. 2009;124(7):1626-36. doi:10.1002/ijc.24116.
14. D'Souza G, Kreimer AR, Viscidi R, Pawlita M, Fakhry C, Koch WM et al. Case-control study of human papillomavirus and oropharyngeal cancer. The New England journal of medicine. 2007;356(19):1944-56. doi:10.1056/NEJMoa065497.
15. Flores-de la Torre C, Hernandez-Hernandez DM, Gallegos-Hernandez JF. Human papilloma virus in patients with epidermoid head and neck carcinoma: a prognostic factor? Cirugia y cirujanos. 2010;78(3):221-8.
16. Kleter B, van Doorn LJ, Schrauwen L, Molijn A, Sastrowijoto S, ter Schegget J et al. Development and clinical evaluation of a highly sensitive PCR-reverse hybridization line probe assay for detection and identification of anogenital human papillomavirus. J Clin Microbiol. 1999;37(8):2508-17.
17. Kleter B, van Doorn LJ, ter Schegget J, Schrauwen L, van Krimpen K, Burger M et al. Novel short-fragment PCR assay for highly sensitive broad-spectrum detection of anogenital human papillomaviruses. The American journal of pathology. 1998;153(6):1731-9. doi:10.1016/S0002-9440(10)65688-X.
18. Kleter B, van Doorn LJ, Schrauwen L, Molijn A, Sastrowijoto S, ter Schegget J et al. Development and clinical evaluation of a highly sensitive PCR-reverse hybridization line probe assay for detection and identification of anogenital human papillomavirus. Journal of clinical microbiology. 1999;37(8):2508-17.
19. Melchers WJ, Bakkers JM, Wang J, de Wilde PC, Boonstra H, Quint WG et al. Short fragment polymerase chain reaction reverse hybridization line probe assay to detect and genotype a broad spectrum of human papillomavirus types. Clinical evaluation and follow-up. Am J Pathol. 1999;155(5):1473-8. doi:S0002-9440(10)65462-4 [pii] 10.1016/S0002-9440(10)65462-4.
20. Tavassoli FA, Devilee P. Tumours of the vagina. Epithelial tumours. In: Andersen ES, Paavonen J, Murnahan M, Östör AG, Hanselaar AG, Bergeron C et al., editors. Pathology and Genetics of Tumours of the Breast and Female Genital Organs. Lyon: IARC Press; 2003. p. 293-301.
21. Smith JS, Lindsay L, Hoots B, Keys J, Franceschi S, Winer R et al. Human papillomavirus type distribution in invasive cervical cancer and high-grade cervical lesions: a meta-analysis update. Int J Cancer. 2007;121(3):621-32. doi:10.1002/ijc.22527.
22. Kreimer AR, Clifford GM, Boyle P, Franceschi S. Human papillomavirus types in head and neck squamous cell carcinomas worldwide: a systematic review. Cancer epidemiology, biomarkers & prevention : a publication of the American Association for Cancer Research, cosponsored by the American Society of Preventive Oncology. 2005;14(2):467-75. doi:10.1158/1055-9965.EPI-04-0551.
23. Van Aar F, Mooij SH, Van Der Sande MA, Speksnijder AG, Stolte IG, Meijer CJ et al. Anal and penile high-risk human papillomavirus prevalence in HIV-negative and HIV-infected MSM. AIDS. 2013. doi:10.1097/01.aids.0000432541.67409.3c.
24. Cornall AM, Roberts JM, Garland SM, Hillman RJ, Grulich AE, Tabrizi SN. Anal and perianal squamous carcinomas and high-grade intraepithelial lesions exclusively associated with "low-risk" HPV genotypes 6 and 11. International journal of cancer Journal international du cancer. 2013;133(9):2253-8. doi:10.1002/ijc.28228.
25. IARC. Human Papillomaviruses. IARC monographs on the evaluation of carcinogenic risks to humans. 2005;90.
